# Micro-Sized pH Sensors Based on Scanning Electrochemical Probe Microscopy

**DOI:** 10.3390/mi13122143

**Published:** 2022-12-04

**Authors:** Muhanad Al-Jeda, Emmanuel Mena-Morcillo, Aicheng Chen

**Affiliations:** Electrochemical Technology Center, Department of Chemistry, University of Guelph, 50 Stone Road East, Guelph, ON N1G 2W1, Canada

**Keywords:** localized pH, microelectrode probe, microsensor, nanosensor, potentiometric sensor, voltammetric sensor, scanning electrochemical microscopy

## Abstract

Monitoring pH changes at the micro/nano scale is essential to gain a fundamental understanding of surface processes. Detection of local pH changes at the electrode/electrolyte interface can be achieved through the use of micro-/nano-sized pH sensors. When combined with scanning electrochemical microscopy (SECM), these sensors can provide measurements with high spatial resolution. This article reviews the state-of-the-art design and fabrication of micro-/nano-sized pH sensors, as well as their applications based on SECM. Considerations for selecting sensing probes for use in biological studies, corrosion science, in energy applications, and for environmental research are examined. Different types of pH sensitive probes are summarized and compared. Finally, future trends and emerging applications of micro-/nano-sized pH sensors are discussed.

## 1. Introduction

Chemical reactions are often strongly influenced by the concentration of hydrogen ions (H^+^) in solution, making its measurement crucial to characterize aqueous solutions [1]. As H^+^ concentration may span many orders of magnitude, it is commonly represented on a logarithmic scale as pH. Many biological mechanisms depend heavily on electrochemical gradients or differences in pH across a membrane to enable their biological functions. Proton transfer plays a key role in biological systems, underlying the functioning of many enzymatic reactions and ion channels. H^+^ concentration greatly influences the function of proteins that act as proton regulators, especially if the pH reaches beyond acceptable working levels. Further, when a cell undergoes inflammation or tumorigenesis, the unhealthy cell acidifies its environment, thus promoting tumor metastasis and contributing to normal cell death. A cancer cell’s local pH plays an important role in cancer progression by modulating its invasiveness and resistance to therapy [2,3,4]. 

Solution pH also has a strong influence on many industrial processes, such as metal electroplating and paint electrodeposition. For example, pH influences the conductivity and current density needed to electrodeposit metallic alloy coatings [5,6], as well as the surface thickness, roughness, resistance to cracking, and corrosion of the coatings [6,7]. The stability of coatings and their resistance to bacterial growth are also influenced by the environmental pH both before and after the deposition of the coating [8,9,10,11].

**Scheme 1:**
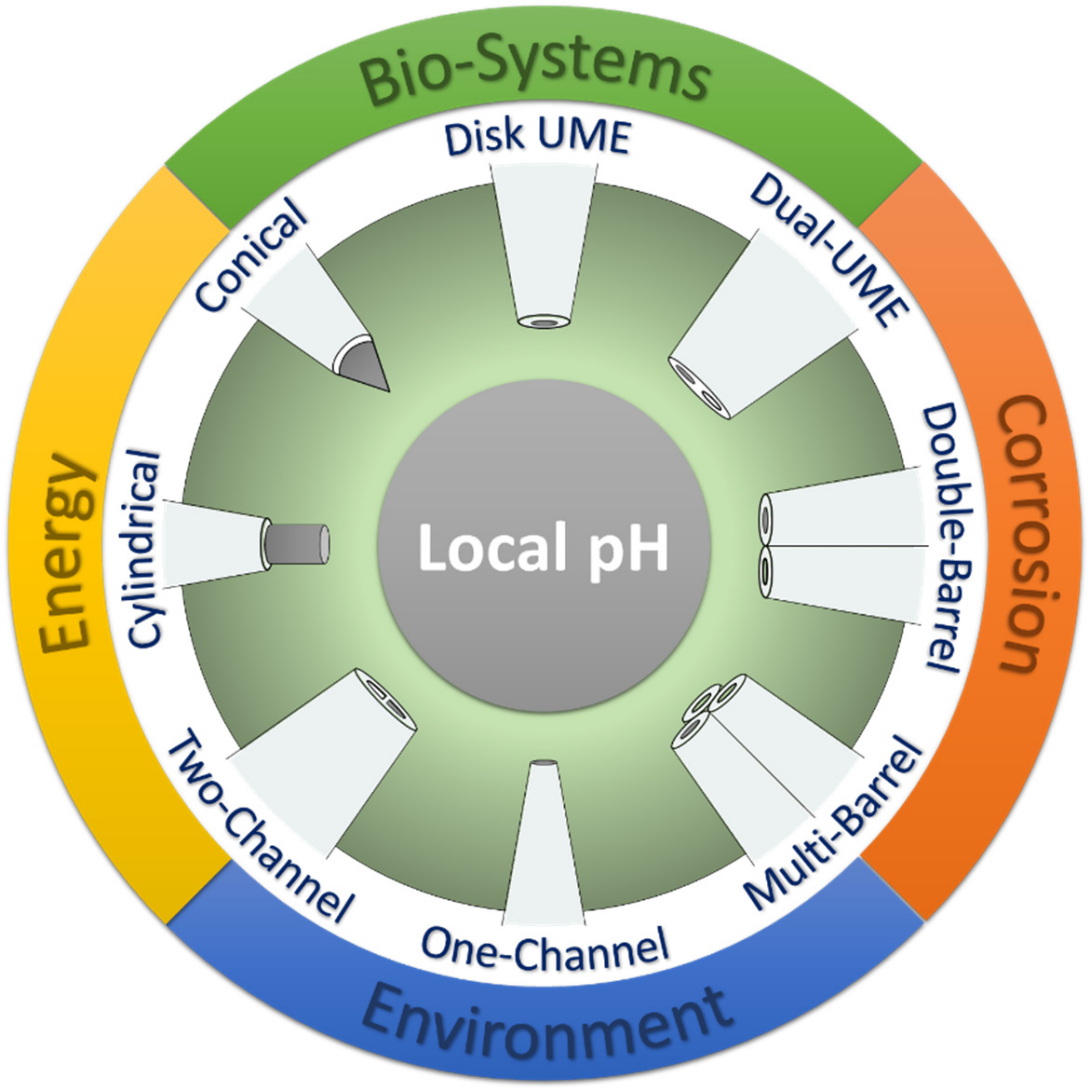
Overview of different types of SECM probes classified by their geometry (disc, conical, cylindrical), number of electrodes (single, double, multiple), and open end (one or two channel micro-/nanopipettes) for the measurements of local pH and applications in biological systems, corrosion science, energy applications, and environmental research.

Local pH measurements can be useful to elucidate and modify the chemical environment, such as in microfluidic applications [12,13,14]. They can also help to decode reaction mechanisms that depend on or affect the local pH. Scanning electrochemical microscopy (SECM) is a type of scanning probe microscopy (SPM) that generates images by scanning a specialized probe over the sample. Using SECM allows the transformation of individual pH measurements into two-dimensional (2D) or three-dimensional (3D) pH images. Electrochemical pH sensors have several advantages over optical and spectroscopic methods: pH is measured directly by sensing either the potential or the current without a pH-sensitive fluorophore, which could potentially affect the solution pH; detection takes place at the electrode tip, allowing for fast and more locally specific pH measurements [15]. SECM also has the advantage of customizable sensing tips. SECM probes have become increasingly miniaturized, and specialized sensing tips (e.g., ultramicroelectrodes (UMEs) and nanoelectrodes (NE)) are important tools for achieving spatially resolved electrochemical images. Scheme 1 presents an overview of the different types of micro-sized pH sensors based on SECM as well as the most common research fields where they have been applied. The probes can be made of various pH-sensing materials by different methods, depending on the needs of the specific final application.

When attempting to generate 2D or 3D pH images using SECM, two key pieces of information are needed: a local pH value and the topography of the associated location on the sample. Like other SPM methods, the SECM probe must also have a method to measure the tip-to-substrate (or tip-to-sample) distance alongside the electrochemical property of interest. Several tip-to-substrate measuring methods exist, including feedback mode of SECM, shear force, and scanning ion-conductance microscopy (SICM), among others. It is important to consider the distance measurement method when designing a pH SECM experiment; this will be further discussed in Section 4.

Since the invention of the first glass pH sensor, scientists have made great efforts to miniaturize and improve the quality, speed, and efficiency of these sensors for reliable, rapid, and increasingly localized pH measurements. Considerable progress has been made in improving sensor accuracy, increasing the active pH range, and extending the stability and longevity of the pH sensing materials, which has broadened their applicability to a wider range of applications. When designing microelectrode- or micropipette-based pH sensors, there are several key parameters that must be taken into consideration: sample type, pH-sensing material, tip-to-substrate distance sensing method, tip size and shape requirements, range, sensitivity, and the speed of both the pH and tip-to-substrate distance measurements.

## 2. pH-Sensing Materials and Their pH-Sensing Mechanisms

While there are many materials and methods for fabricating pH-sensitive microelectrodes, research has shown that metal oxide (MOx) sensors have unique and highly desirable properties. For example, MOx-based pH-sensitive materials typically have high surface-to-volume ratios, allowing for highly sensitive pH measurements with good selectivity for H^+^ over other small cations. They tend to have good catalytic activity in pH-measuring reactions, yet they are biologically inert. These properties, along with their fast response, extended lifetimes, and stability in a variety of atmospheres, make them suitable for a variety of applications [16]. They are usually easily deposited on different substrates, which makes them good candidates for applications in novel microelectrode arrays [17,18].

Among MOx materials, iridium oxide (IrOx)-based pH sensors exhibit fast response and high stability over a wide range of pH, temperature, and pressure. They can also function in aqueous and non-aqueous solutions, including aggressive environments such as corrosive media. Like other MOx materials, the applicability of IrOx pH sensors depends heavily on how they are deposited onto the ultra-microelectrode (UME) tip [16,19,20]. There are different methods for depositing IrOx, including electrochemical deposition, reactive sputtering with an iridium target, and thermal decomposition of iridium precursors [19,21,22]. A popular and comparatively simple method to coat a conductive surface with IrOx is the anodically deposited iridium oxide film (AEIROF) procedure first introduced by Yamanaka [22] in 1989, which uses an alkaline aqueous solution of iridium chloride to electroplate an electroactive surface. The pH sensing mechanisms of MOx, in particular IrOx, have been extensively studied [16,23,24,25]. Generally, metal/MOx-based pH-sensing electrodes may reach the following equilibrium [1]:

M_x_O_y_ + 2y H^+^ + 2y e^−^ ⇔ x M + y H_2_O (1)

Multiple proposed pH-sensing mechanisms are possible, but the most likely is the oxygen intercalation mechanism proposed by Fog and Buck [23] as it closely matches the known non-stoichiometric oxygen content of these metal oxides. The pH-sensing mechanism of IrOx is likely due to the reversible electrochemical reaction of Ir(III) and Ir(IV) [26]:

2 IrO_2_ + 2 H^+^ + 2 e^−^ ⇔ Ir_2_O_3_ + H_2_O (2)

The mechanism may be affected by the environment being hydrated or non-hydrated. While much work has been conducted to uncover the exact mechanism, complex reactions involving other hydrated or anhydrous iridium oxides may occur. Similar mechanisms have also been proposed for other metal oxides, such as ruthenium oxide (RuOx), antimony oxide (SbOx), cerium oxide (CeOx), and tin oxide (SnOx) [16,20,23,24]. Antimony oxide pH sensing electrodes are often used in pH-SECM applications. Like other MOx materials, although the exact mechanism is unknown, it is believed that multiple redox reactions may be involved, but the main detection redox reaction is the following [1,27]:

Sb_2_O_3_ + 6H^+^ + 6e^−^ ⇔ 2Sb + 2H_2_O (3)

However, research on antimony oxide pH electrodes has also shown that continuous adsorption of oxide species onto the surface increases the coating thickness and reduces the accuracy of the sensor for pH measurements [27,28].

Like MOx materials, polymers such as polyaniline (PANI) and syringaldazine (Syr) have also gained popularity in pH sensing in recent years [29,30,31,32,33,34,35,36,37,38]. PANI is a promising solid-state pH sensor due to its good conductivity, low cost, biocompatibility, high stability, and well-established electrochemical polymerization procedure. PANI may undergo two redox processes—protonation during reduction and deprotonation during oxidation. The favorability of the two processes strongly depends on the pH of the aqueous electrolyte, which allows sensitive pH determination [30,31]. Those pH-sensitive polymers, such as syringaldezine, are insoluble in water, thus making them less likely to desorb from the electrode when used in aqueous solutions. Syringaldezine, similar to PANI, can also easily and readily undergo electrochemical conversion between its two reversable forms, involving two protons and two electrons, thus making it sensitive to the concentration of protons in solution. The pH change may be monitored by the shift in peaks observed in cyclic voltammetry, where a more positive peak indicates a lower pH value [36,37].

## 3. Sensor Fabrication

**Figure 1:**
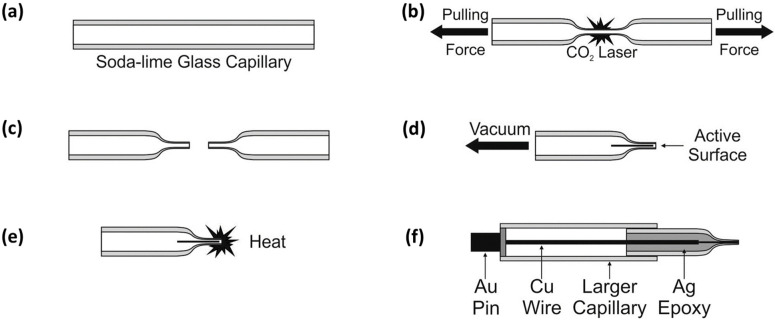
UME fabrication technique [39]: (**a**) bare soda-lime glass capillary, (**b**) heating the capillary with a CO_2_ laser and pulling to form a sharp tip, (**c**) the two separated micropipette ends, (**d**) an electroactive wire is inserted into the micropipette and maneuvered to the tip before a vacuum tube is attached to the open end, (**e**) the electroactive wire is sealed in position under vacuum with a heating coil, and (**f**) the UME is finalized by adding the connective wires and the outer protective glass capillary. Adapted from ref. [39]. Copyright (2015) with permission from the American Chemical Society.

Almost all of the methods currently in use for fabricating SECM sensor probes, whether based on micropipettes and/or microelectrodes, involve heat-pulled glass pipettes as the backbone onto which the rest of the sensor components are assembled. While there are a variety of methods to fabricate these sensing probes, a typical procedure for making a micropipette is illustrated in Figure 1a–c, and a typical procedure for preparing a microelectrode is illustrated in Figure 1d–f [39].

### 3.1. Micropipette Glass Backbone Fabrication

Typically, a heating coil- or laser-based pipette puller is used to heat and pull apart a single glass capillary made of soda-lime, borosilicate, or quartz glass until it is split in half, resulting in two micropipettes, as shown in Figure 1a–c. The geometric properties of the formed pipettes can be controlled and tuned by adjusting the parameters of the micropipette puller. These micropipettes are now ready to be modified with a pH-sensing material either on the inside or outside surface using sputtering or other methods.

### 3.2. Microelectrode Glass Backbone Fabrication

A similar procedure is used to prepare the glass supports for microelectrode-style probes. After using the pipette puller to obtain the two glass pipettes, a small diameter electroactive wire (typically gold, platinum, or carbon-fiber) is inserted into the open end and then carefully maneuvered to the sharp end with the help of a micromanipulator or other methods, as shown in Figure 1d. Alternatively, the wire may be inserted into the glass capillary before it is pulled apart. The wire is then fixed into position using a small heating coil to collapse the glass around the wire at the pipette tip under vacuum (Figure 1d,e). The connecting wire is then inserted through the open back end of the pipette and attached to the smaller wire using conductive silver epoxy, as shown in Figure 1f. A general-purpose SECM sensor probe can be made by sanding and polishing the pipette tip to expose the tip of the electroactive wire. Alternatively, depending on the required tip geometry (disk, needle, ring, etc.), other methods, such as ion beam or electrochemical etching, can be employed to optimize the portion of the electroactive wire that will later be modified. Next, the electroactive surface is functionalized with a pH-sensitive material. In the next sections, steps for modifying a general tip into a pH sensor, along with methods for implementing tip-to-substrate measurements, will be presented.

### 3.3. Microelectrode-Based pH Sensors

**Figure 2:**
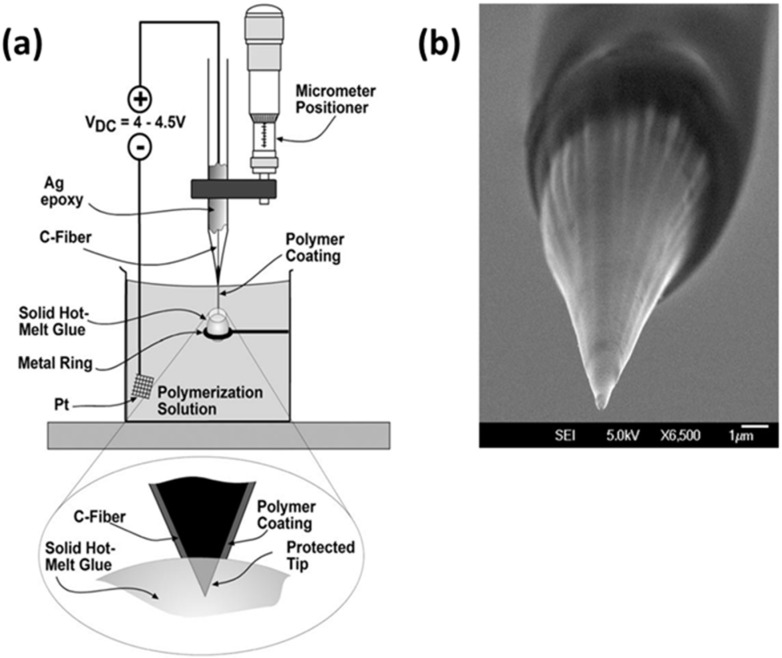
pH SECM microelectrode fabrication and protection: (**a**) Schematic diagram for the tip protection step right before electropolymerization of poly(oxyphenylene) onto the rest of the electrode, (**b**) SEM micrograph of the poly(oxyphenylene) protected carbon fiber UME. Reprinted from ref. [42]. Copyright (2006) with permission from John Wiley and Sons.

Once the glass backbone of the microelectrode has been prepared, it needs to be coated with a pH-sensitive material. There are many methods used to achieve this, and only a few notable methods are highlighted here. A facile procedure for fabricating pH-sensitive microelectrodes was reported by Khani and Wipf [40] in which a carbon fiber (CF) was electrochemically etched, its tip was then shielded, and poly(oxyphenylene) was electropolymerized onto the unshielded portion of the tip to insulate it, as illustrated in Figure 2. With this procedure, only the very tip of the needle-like ultramicroelectrode was exposed, making it ready for electrodeposition of IrOx using an adapted procedure from Pikulski and Gorski [41]. Briefly, a three-electrode set up was used with the CF-UME as the working electrode, while an Ag/AgCl wire served as the reference electrode, and a Pt wire served as the counter electrode. The electrodeposition solution was 0.2 mM Na_3_IrCl_6_ and 0.1 M KNO_3_ solution, which was previously heated up for 3 h and cooled to encourage the aquation of the iridium complexes. In that solution, 50 cyclic voltammetric cycles from −0.2 V to 1.2 V were run, followed by holding the potential at 0.6 V for 5 min, resulting in the formation of IrOx onto the exposed area of the CF-UME tip, thus creating a pH-sensitive UME. More details about this design can be found in references [40,41,42].

Another good approach for the fabrication of probes capable of performing two types of measurements (pH and tip-to-sample distance) was proposed by Santos et al. [43], where a single disk-shaped electroactive surface was covered with intentionally defective layers of IrOx. The porosity and defects of the IrOx film are intended and required for this method to work, since this correctly allows the species in the solution to reach both the IrOx to perform the pH measurements and the underlying gold electrode for use in distance control in SECM feedback mode. The above was achieved by encapsulating a 25 μm diameter gold wire in a borosilicate glass capillary and pulling it apart in a similar procedure as the one described in the beginning of this section. Once the bare Au electrode was fabricated, the IrOx electrodeposition solution was made according to a procedure that was adapted from earlier work by Yamanaka and Elsen et al. [22,44]. A three-electrode cell system was used for the electrochemical deposition, with the gold electrode as the working electrode, Ag/AgCl for the reference electrode, and a platinum wire as the counter electrode. The first step was to hold the potential at 0.8 V for 600 s, then cyclic voltammetry in the range between 0 to 0.8 V was run for 5 cycles or more, with thicker and more homogenous coverage of the Au surface as the number of cycles increased. Unfortunately, methods like these that rely on the same surface to do separate measurements may result in competition between the two measurement types, in which one measurement would have to be sacrificed in order to improve the other. There may also be some stability and longevity drawbacks for this design. If the film was made thicker, it would improve the stability of the IrOx film on the gold electrode; however, the thicker film might also restrict the species in the solution from reaching the underlying Au surface, thus making the tip-to-substrate measurements less accurate and often resulting in crashing the tip to the sample.

Monteiro et al. [45] designed a probe that consisted of adsorbing an organic self-assembled monolayer (SAM) of 4-nitrothiophenol (4-NTP) onto a disk gold UME, which was then converted into the redox couple hydroxylaminothiophenol (4-HATP)/4-nitrosothiophenol (4-NSTP). Briefly, a gold wire was encapsulated in glass, cleaned, and polished using similar procedures as described above. Once the bare gold UME was prepared, it was modified by immersing it into a 4-NTP/ethanol solution for 20 min, which spontaneously resulted in the irreversible adsorption of a 4-NTP monolayer onto the Au surface through the thiol anchor group. After the UME was rinsed, it was transferred into a 0.1 M H_2_SO_4_ solution and polarized in the range of 0.1 to −0.25 (vs. Ag/AgCl) in order to convert the 4-NTP into 4-HATP and 4-NSTP species to make the probe sensitive to pH changes. To make pH measurements, the reversable oxidation of the 4-HATP to 4-NSTP species through the transfer of two protons and two electrons is monitored using cyclic voltammetry [45,46,47].

### 3.4. Micropipette-Based pH Sensors

A popular pH sensor design is the use of an ion-selective cocktail for the detection of local pH changes. There are a variety of methods used to fabricate such probes, including both single- and double-barrel pH sensors, and a variety of different ion-selective cocktails are capable of detecting local concentrations of protons. For example, Joshi et al. [48] developed this type of pH sensor based on an H^+^ ion-selective cocktail consisting of 5% proton ionophore I, 2% potassium tetrakis(4-chlorophenyl)borate, 30% 1-(2-nitrophenoxy) octane (NPOE), 3% poly(vinyl chloride) (PVC), 60% Vulcan carbon powder, and 500 μL of tetrahydrofuran (THF). Their procedure started with pulling a borosilicate glass pipette apart using a similar procedure as described above. Once a glass backbone was created, their H^+^ ion-selective cocktail was mixed and backfilled into the pipette from its back end, then pushed further into the tip using a copper wire, before adding a mixture of 5% Vulcan carbon in dioctyl sebacate (DOS). The electrical connection was made using a copper wire, which was stabilized in position using epoxy. Another example of the use of ion-selective pH sensors is the work published by Etienne et al. [7], in which a different commercially available ion-selective cocktail was chosen: pH-ionophore II–cocktail A. A similar procedure was employed to pull apart the borosilicate glass capillary to obtain a micropipette. The micropipette was then polished on a microelectrode beveller, and hexamethyldisilazane was used to silanize the inner cavity of the micropipette before filling it with the ion-selective cocktail, then backfilling it with 0.1 M KCl solution on top for use as the internal electrode.

**Figure 3:**
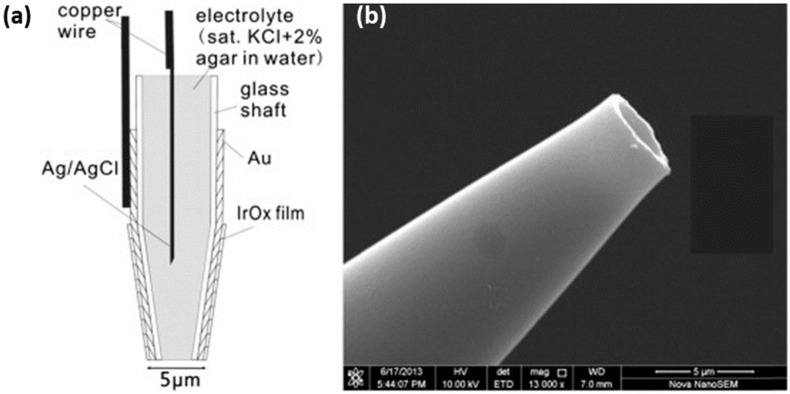
(**a**) Schematic of the coaxial pH sensor. (**b**) SEM micrograph of the ~5 µm tip of the pH sensor without the reference electrode. Reprinted from ref. [49]. Copyright (2014) with permission from Elsevier.

Huang et al. [49] designed a miniaturized coaxial pH sensor that encapsulated the Ag/AgCl reference electrode inside of the pipette cavity of the sensor, while the gold and pH-sensitive IrOx were deposited on the outside of the sensor. A thin layer of Au was deposited on the outside of the pipette by RF magnetron sputtering. Conductive silver epoxy paste was used to attach a copper wire to the Au layer. Agar and KCl were mixed, boiled, and then inserted into the pipette from its back end using a syringe. While the solution was still hot, an Ag/AgCl wire was inserted into the back of the pipette, which was then sealed using silicon rubber. Finally, to make the tip of the pipette sensitive to pH, IrOx was electrodeposited onto the Au film using a modified Yamanaka procedure [21,22]. The resultant pH sensor is illustrated in Figure 3a and an SEM image of the tip of the pH sensor is displayed in Figure 3b [49], further confirming the success of the fabrication.

**Figure 4:**
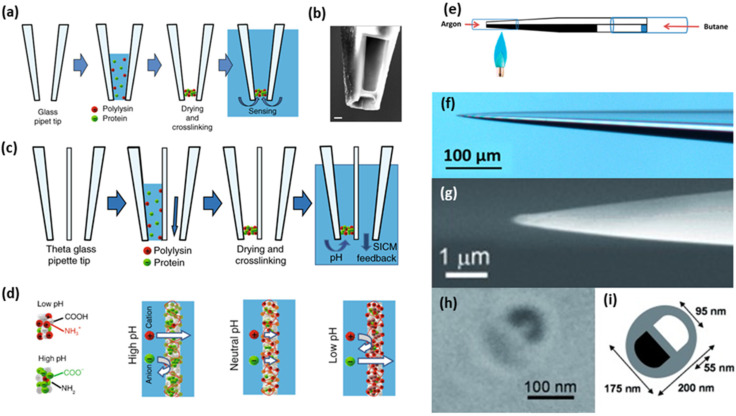
Fabrication and modification steps for both single- and double-barrel pH sensing probes. (**a**) Self-assembly of poly-l-lysine and glucose oxidase (PLL/GOx). (**b**) SEM image after FIB milling (scale bar = 500 nm), showing the thin membrane deposited at the end of the pipette’s tip. (**c**) Self-assembly of PLL/GOx for a double-barrel pH sensor. (**d**) The proposed mechanism for pH sensing using the PLL/GOx nanomembrane. (**e**) Schematic of the procedure for pyrolytic deposition of butane gas into carbon on the inside of one of the double-barrel pipette openings. (**f**) Side view optical image. (**g**) Side view SEM micrograph. (**h**) Bottom view SEM micrograph. (**i**) Schematic of the carbon-based pH sensor before deposition of iridium oxide. Images (**a**–**d**) were reprinted from ref. [2] under CC-BY 4.0 license. Images (**e**,**f**) were adapted from ref. [50]. Copyright (2013) with permission from the American Chemical Society. Images (**g**–**i**) were adapted from ref. [51]. Copyright (2011) with permission from John Wiley and Sons.

Zhang et al. [2] developed two versions, single-barrel and double-barrel, of a pH sensitive nanopipette based on self-assembled zwitterion-like nanomaterials, which may favor the permeability of anions at low pH and cations at high pH, as illustrated in Figure 4. The single-barrel pH-sensing nanoprobe was fabricated using a similar procedure as described above for pulling a borosilicate glass capillary to the required size for a nanopipette, as seen in Figure 4a. Glucose oxidase (GOx) denatured at 70 °C for 10 min was then dissolved in 0.01% poly-l-lysin (PLL) at a concentration of 0.4 mg/mL to make a hydrogel, which provided the positively charged residues through the amines of PLL and the negatively charged residues through GOx. The hydrogel was drawn up into the pulled glass pipette through capillary action to a height of around 1 mm from the opening of the tip. The hydrogel was then used to self-assemble the residues onto the opening of the pipette through drying of the sensing material nanomembrane. The vapor of glutaraldehyde (25% *v*/*v*) was used to slowly crosslink the hydrogel residues inside the glass pipette, as seen in Figure 4a. Strong covalent bonds were expected to form between the amino groups left by the PLL, GOx, and glutaraldehyde vapor, forming a robust nanomembrane (~200 nm) capable of sensing local pH at the tip of the pipette. The remaining steps included cleaning the probe with KCl (100 mM pH 7.0) to remove any remaining glutaraldehyde and unreacted or unlinked residues. To prepare the double-barrel nanopipette, a similar procedure was followed for making the glass backbone and the rest of the probe, except for the use of “Blu-tack” to block one of the two barrels. A small pressure of ~200 kPa was applied to the second barrel to resist drawing up the PLL/GOx hydrogel through capillary action. The second pipette barrel would remain open for use in SICM tip-to-substrate distance measurements. The single-barrel probe was capable of sensing both localized pH as well as tip-to-sample distance measurements through the linear ionic response at the same single pipette tip during its approach to the sample. However, while measuring the SICM ionic current as a feedback control signal (as detailed in Section 4.4), along with the localized pH, the single-barrel sensor is sensitive to any extracellular pH changes and can introduce some unexpected artifacts, which appear as a ball-like shape at the H^+^ source. This can be remedied with the use of the dual-barrel version of the pH probe or, alternatively, by making a height measurement with the single-barrel probe relatively far away from the H^+^ source, and then constant-height mode can be used to image the rest of the sample [2].

Nadappuram et al. [50] proposed a simple procedure to build pH probes based on SECM-SICM, using a procedure similar to the one described by Takahashi et al. [51]. The sensor is a double-barrel nanopipette that is capable of measuring localized pH through one of the barrels using a pyrolytically deposited carbon electrode that has been modified with IrOx by electrodeposition. The other side of the electrode is left open and used to measure tip-to-substrate distances. To achieve this, Nadappuram et al. pulled apart a quartz theta (i.e., double-barrel) capillary and blocked one of the pipette openings with “Blu-Tack” putty, while a butane gas tube was attached to the other opening. In an argon atmosphere, butane was supplied through the tube, and the tip of the probe was heated using a torch to induce pyrolytic decomposition of butane, resulting in the deposition of carbon inside one of the pipette’s tips. A simple schematic of this procedure is illustrated in Figure 4e, while the probe’s optical and SEM side-image micrographs are presented in Figure 4f and g, respectively. An SEM micrograph and illustration of the bottom of the tip of the sensor are also presented in Figure 4h and i, respectively. Next, a wire was connected to the deposited carbon through the back of the pipette and hydrated IrOx was deposited onto the carbon-filled tip of the pipette by electrodeposition using similar methods as described previously [22], which was achieved by holding a constant potential at 0.68 V (vs. Ag/AgCl QRCE in 0.1 KCl) for 600 s [50].

## 4. Positioning Methods

Successful implementation of a pH sensor requires precise positioning of the tip above the sample’s surface. This can be achieved through different means, including qualitative and quantitative approaches. The simplest method used to place the sensor tip near the substrate is by observing it with the aid of a microscope [52,53,54]. This procedure is useful for UME diameters ≥ 10 µm. Although it provides a reasonable approximation, the true tip-to substrate distance remains unknown. For smaller UME diameters, the optical approach becomes challenging to apply. Liu et al. [34] utilized a charge-coupled device to establish the distance between the UME and the substrate. It must be noted that the methodology worked to place the tip at a far distance from the substrate (200 μm). Another simple way to position the tip is by monitoring its potential while approaching the sample and stopping when a signal “jump” is observed [55]. The main disadvantage is that the tip could be damaged and negatively affect the pH measurements.

The most popular method, by far, is the feedback mode of SECM. However, this method still has two main drawbacks: the need of a redox mediator and the convolution of topography/reactivity in the current signal. Alternatively, other methods, such as shear force (SF) sensing, alternating current-scanning electrochemical microscopy (AC-SECM), and scanning ion-conductance microscopy (SICM), have been successfully applied in the literature. These methods have been demonstrated to provide reliable strategies for fine control of the tip-to-substrate distance, and at the same time, they overcome the issues experienced by the feedback mode. Some of these methods can also be useful when imaging rough surfaces or samples with 3D geometry [56]. Although there are other positioning methods, this review presents exclusively those used alongside localized pH measurements. In the following sections, a more detailed description of each method will be presented.

### 4.1. Feedback Mode of Scanning Electrochemical Microscopy

Feedback mode is the native mode of SECM, which employs a three-electrode configuration: the UME as the working electrode, a reference electrode, and a counter electrode. In brief, a redox reaction occurring at the biased UME induces a steady state current when the probe is far from the substrate. The relationship between the diffusion limited steady-state current, *i_T,__∞_*, redox mediator parameters, and the geometry of the UME is described as follow [57]:

(4) $$i_{T,\infty }=4nFDCr_{UME}b$$

where *n* is the number of electrons involved in the redox reaction, *F* is the Faraday constant, *D* is the diffusion coefficient of the redox mediator, *C* is the concentration of the redox mediator, *r_UME_* is the radius of the true electroactive surface of the UME, and *b* is a geometric constant that depends on the RG value of the UME (ratio between *r_UME_* and the radius of the insulting sheath, *rg*). For large RG values (>10), *b* is equal to 1. However, there is an increase in the diffusion-limited current for small RG values (RG < 10), where the *b* value can be calculated to account for the *i_T,∞_* enhancement [58].

**Figure 5:**
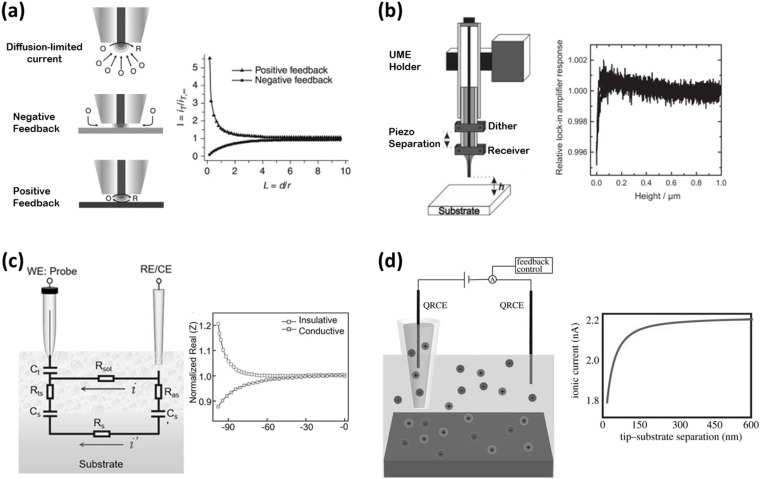
Probe-positioning methods for pH sensors and examples of their approach curves. (**a**) Hemispherical diffusion-limited current and pure feedback cases of scanning electrochemical microscopy. Adapted from ref. [74]. Copyright (2012) with permission from Elsevier. (**b**) Shear force sensing in the two piezo dither/receiver arrangement. Adapted from refs. [75,76]. Copyright (2013) and Copyright (2014) with permission from Elsevier. (**c**) Alternating current-scanning electrochemical microscopy and the equivalent circuit for the overall impedance response. Adapted from ref. [77]. Copyright (2019) with permission from John Wiley and Sons. (**d**) Representation of the operation principle of the scanning ion-conductance microscopy. Adapted from ref. [78]. Copyright (2017) with permission from The Royal Society.

Figure 5a shows the two pure feedback cases and examples of their corresponding approach curves. The value of the current changes when the tip is close to the substrate’s surface, depending on the reactivity of the latter. For example, if the UME approaches to an insulating substrate, the diffusion of the redox species is physically blocked and therefore the current decreases. On the other hand, if the UME approaches a conductive substrate, redox species are regenerated at the substrate’s surface, enabling a feedback loop and thus, an increase in current. These alterations in the steady-state current are known as negative feedback (*i_NFB_* < *i_T,__∞_*) and positive feedback (*i_PFB_* < *i_T,__∞_*), respectively. Once the experimental approach curve is recorded, it can be fitted to analytical expressions to quantitatively establish the tip-to-substrate distance [58].

The feedback mode has been widely employed for positioning the UME prior to pH measurements [28,35,36,37,43,48,59,60,61,62,63,64]. There are two ways to accomplish this: one is using a single-electrode probe, whereas the other is using a dual-electrode probe. The only requirement is that the probe be able to perform both amperometric and potentiometric measurements. Examples of using a single-electrode probe include UMEs made of Sb/SbOx [59], W/WOx [63], and Pt/IrOx [43]. In order to achieve dual function in this type of UME, the oxide film must be defective to allow the redox reaction to occur at the tip [43]. Alternatively, dual-electrode probes can be used to overcome issues with using a defective film. In this case, one electrode is used in amperometric mode, whereas the other electrode is modified to work as a potentiometric pH sensor. This approach has gained popularity and is widely reported in the literature [28,35,60,62,64,65].

### 4.2. Shear Force Sensing

Another technique used to position the probe is so-called shear force control. This method is based on sensing the hydrodynamic forces between the oscillating probe and a surface in close proximity. The probe is stimulated through lateral vibrations (by applying an AC voltage to a piezoelectric plate) while measuring the amplitude/phase of the oscillations at a certain frequency. When the tip-to-substrate distance reaches tens to hundreds of nanometers, shear force damping occurs, and a change in the amplitude/phase is detected. There are a few ways of measuring this shear force damping. The first method involves detecting the frequency of damping by means of a laser and split photodiode [66]. The second method involves a tuning fork fixed to the probe, with the resonance peak being sensitive to the changes in shear force [67]. Finally, the most practical method is to use two piezo crystals, one as a dither and the other as a receiver, where the latter directly measures the oscillations produced by the former [68].

Figure 5b presents the shear force setup with the dither-receiver piezo elements. In the approach curve, the amplitude value for a specific frequency sharply decreases when the tip is very close to the substrate. The greatest advantage of the shear force method is that it provides independent control of the tip-to-substrate distance, which is not affected by the electrochemical reactivity of the substrate nor the electrical properties of the probe. Therefore, both topography and pH data can be gathered simultaneously. This is very useful when employing potentiometric sensors, as they could be destabilized if current passes thought them. Therefore, coupling shear force with local pH sensing has been successfully carried out in the literature [7,69,70,71,72,73].

### 4.3. Alternating Current-Scanning Electrochemical Microscopy

The use of an alternating current (AC) signal has also been applied to position SECM probes [79]. In this technique, a low-amplitude oscillating potential is imposed to the UME while its alternating current response is acquired. Therefore, the localized impedance of the system can be measured. AC-SECM works well in low-conductivity electrolytes and does not require the presence of a redox mediator. By using a low amplitude, electrochemical modification of the pH sensor can be avoided. Figure 5c shows the equivalent circuit for modeling the overall impedance response [77]: *R_sol_* represents the solution resistance between the tip and auxiliary electrodes; *R_s_* is the substrate’s resistance; *R_ts_* is the solution resistance between the tip and the substrate; *C_t_* is the capacitance of the tip; *C_s_* is the capacitance of the substrate right below the tip; and *R_as_* and *C_s_’* are associated with the resistance and capacitance between the substrate and the auxiliary electrodes, respectively. Considering an electrolyte with low conductivity and an insulating substrate, the equivalent circuit can be simplified to *R_sol_* in series with *C_t_*, where *C_t_* is considered insensitive to tip-to-substrate distance. Therefore, the impedance becomes a function of only the solution resistance. The resistance in bulk solution is a function of solution resistivity, *ρ*, and the radius of the UME, as presented below:

(5) $$R_{sol}=\rho /4r_{UME}$$

When the tip approaches the insulating substrate, the solution resistance increases due to blockage of the AC current pathway. By normalizing the solution resistance measured, the recorded approach curve can be fitted with negative feedback theory [79].

If the substrate is conductive, the AC current encounters an alternative pathway through the substrate. In this case, the equivalent circuit is more complex. Furthermore, if the substrate is connected to the system and held at a constant potential, the equivalent electrical circuit becomes challenging to construct [80]. Hence, the main strategy followed in literature is to approach pH sensors over an insulating region of the substrate [81,82,83,84].

### 4.4. Scanning Ion-Conductance Microscopy

Positioning of micropipette-based probes has been achieved using scanning ion-conductance microscopy (SICM). In this approach, the ionic currents flowing between an electrode inside a micropipette and a secondary electrode placed in bulk solution are measured and used as a feedback signal [85]. Figure 5d displays the principle of operation of SICM and an example of an approach curve.

The value of the ionic current, *i_∞_*, depends on the applied potential, *E*, and the resistance of the pipette (*R_p_*), which is a function of electrolyte conductivity and the geometric parameters of the micropipette [86]:

(6) $$i_{\infty }=E/R_{p}$$

The ion current is highly sensitive when the tip-to-substrate distance is similar to the radius of the micropipette. As the separation distance becomes smaller, the ionic conductance current sharply decreases because the flow of the ions is hindered by the proximity of the substrate. This characteristic distance-dependent ion current has been exploited to measure the topography of a wide variety of different samples [87]. One important aspect of SICM is that the resolution is mostly dependent on the size of the micropipette tip opening, which makes it relatively easy to reach the nanometer range. Combining SICM with pH sensors has enabled not only positioning of the probe, but also acquiring simultaneously topographic and pH information. For such purpose, both single-channel [33,88] and double-channel [2,50] pipettes have been successfully demonstrated in the literature.

### 4.5. Other Positioning Methods

There are a couple of other positioning methods that are less commonly used than those highlighted above. The first method is the capacitive approach, which is based on determining the tip−substrate capacitance in air [89]. The main feature of this methodology is that it does not require electrolyte. In brief, an AC potential is imposed on the substrate, while the current at the UME is followed with a low-noise preamplifier, and the capacitance is calculated using a lock-in amplifier [45]. In this scenario, the tip–sample capacitance can be described as follows [89]:

(7) $$C_{par}=\varepsilon_{0}A/d,$$

where *ε*_0_ is the permittivity of air; A is the area of the UME; and *d* is the separation distance between the tip and substrate. When the tip gets closer to the substrate, the capacitance suddenly increases. This capacitive approach was successfully used by Monteiro et al. [45,46,47] to position a voltammetric pH sensor before measurements.

The second method relies on using the concentration profile obtained experimentally and fitting it to the theoretical profile according to the substrate’s geometrical and electrochemical properties [90]. The main requirement for this procedure is that the system must be in a steady state, as the fitting is calculated using Fick’s second law of diffusion [91]. The other important assumption to be made is that the redox species are stable and not involved in a homogeneous reaction in solution [92]. Additionally, the tip affects the concentration profile by introducing both shielding and convection effects as it moves and gets closer to the substrate. All the aforementioned drawbacks restrict a wide application of this method.

## 5. Applications

In the previous sections, we highlighted the fabrication and positioning of micro-/nano-sized pH probes. Some recent work focusing on localized pH measurements using pH probes in the fields of biology, corrosion, energy, and the environment will be described below.

### 5.1. Biological Systems

pH measurements in live systems are important as even small changes at the intracellular and/or extracellular level can dramatically affect numerous biological functions. The pH can also influence the activity of microorganisms and the physiological response of the human body. The use of pH probes for localized pH measurements in biological systems has served as a powerful tool to study bacteria, human cells, and other microorganisms [2,28,37,48,64,65,88]. For example, Harris et al. [65] monitored urea hydrolysis and subsequent calcification processes induced by a *Sporosarcina pasteurii* biofilm in real time. It was found that the pH near the surface of the biofilm increased from 7.4 to 9.2 within 2 min, whereas the Ca^2+^ concentration decreased from 85 to 10 mM within 10 min. The CaCO_3_ precipitation caused an increase of 50 μm in biofilm height after 4 h. The authors concluded that bacterial enzymes promoted a fast urea hydrolysis process and that CaCO_3_ formation/precipitation was the rate-limiting step.

**Figure 6:**
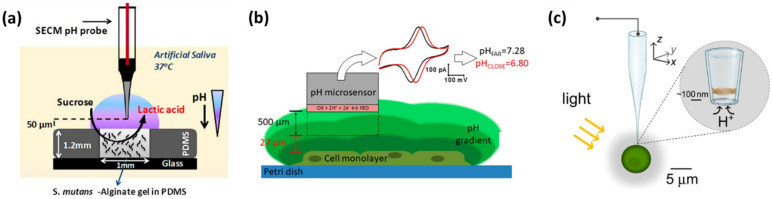
(**a**) Application of a potentiometric pH sensor to obtain a 3D image of pH distribution in the *S. mutans* biofilm. Reprinted from ref. [48]. Copyright (2017) with permission from American Chemical Society. (**b**) Schematic representation of the voltametric pH microprobe for measuring pH gradients in the extracellular space of mammalian cells. Adapted from ref. [37]. Copyright (2018) with permission from the American Chemical Society. (**c**) Scheme showing the pH measurement of the phycosphere of marine green algae *Chlamydomonas concordia* RCC1 and the effect of light/dark. Adapted from ref. [88] under CC-BY 4.0 license.

In another study, Joshi et al. [48] evaluated the microbial metabolic exchange between two bacterial species, *Streptococcus gordonii* and *Streptococcus mutans*. It was shown that H_2_O_2_ generated by *S. gordonii* was inhibited by the lactic acid produced by *S. mutans*. Figure 6a displays the application of pH sensors in 3D mapping, showing that the chemical environment was initially dominated by *S. gordonii* while the buffering capacity of the saliva was still valid (∼pH 6.0−7.2) and that eventually, *S. mutans* took control by decreasing the local pH (≤5.0). Figure 6b illustrates the investigation of pH gradients in the extracellular space of cancer cells and normal cells carried out by Munteanu et al. [37]. The authors observed that cancer cells (HT-29) subjected to hypoxia displayed a more acidic (>0.4 pH units) extracellular environment than normal (HEK-293) or normoxic cancer cells. Work conducted by Liu et al. [88] evaluated the phycosphere pH of marine microorganisms subjected to different environmental conditions. Figure 6c displays the application of a micropipette-based pH sensor to measure the phycosphere pH of single phytoplankton cells (~5 μm diameter) under consecutive light/dark cycles. It was demonstrated that the pH in the phycosphere was consistently different from that of bulk seawater, which challenged the previous assumption on both having the same pH. Zhang et al. [2] employed pH nanoprobes to measure dynamic changes in pH gradients in breast cancer MCF7 cells at the single-cell level. The authors achieved pH mapping with high spatiotemporal resolution, which revealed that the peri-cellular environments of melanoma and breast cancer cells display tumor heterogeneity.

Song et al. [28,64] and Xiong et al. [28,64] used a potentiometric dual-microelectrode to monitor the extracellular pH of MCF-7, HeLa, and HFF cells under electrical stimulation. Their studies showed that by increasing the stimulation potential, the extracellular pH decreased due to the cell membrane becoming more permeable, and that the extracellular pH of cancer cells was lower than that of normal cells. Aref et al. [93] applied a potentiometric pH nanosensor for intracellular measurements, revealing the pH gradient from the extracellular environment to the intracellular environment of a single PC12 cell, as well as variations in its intracellular pH after administration of the drug cariporide.

### 5.2. Corrosion

Corrosion processes involve anodic metal dissolution often accompanied by a cathodic reaction, which produces a local increase in pH. Moreover, metal ions released during corrosion may cause hydrolysis reactions and acidify the surrounding environment [94]. Consequently, it is evident that analyzing localized pH changes can help gain a better understanding of corrosion mechanisms and help to develop corrosion mitigation strategies.

**Figure 7:**
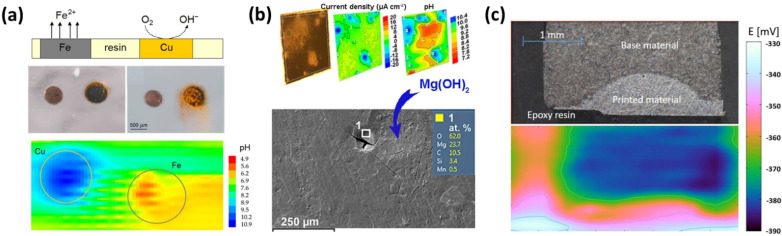
(**a**) Depiction of the galvanic corrosion process of a Cu-Fe pair and its associated local pH map. Adapted from ref. [97]. Copyright (2016) with permission from Elsevier. (**b**) Localized currents and pH maps accompanied with optical and SEM images of the MA8 Mg alloy after being exposed to MEM solution Adapted from reference [100]. Copyright (2020) with permission from Elsevier. (**c**) Distribution of pH at the 3D-printed plate/base material boundary exposed to corrosive medium. Reprinted from reference [63]. Copyright (2020) with permission from John Wiley and Sons.

The initial application of pH microsensors in corrosion research was performed with galvanic couples of pure metals, such as Zn-Fe pairs, as model systems [59,94]. In another study, Filotás et al. employed double-barrel electrode assemblies to explore galvanic corrosion of Zn-Fe [95], Zn-Cu [96], and Cu-Fe couples [97]. Figure 7a displays the reactions taking place, optical images, and the pH map of the Cu-Fe galvanic corrosion process. Further application of local pH sensing includes the evaluation of metallic coatings. Lowe et al. [98] modelled the cut edge corrosion of metallic coatings (Zn, 55% Al–Zn and solid solution Al–40% Zn) on steel. Their results showed that pH decrease caused by steel corrosion, along with the buffering effect of Al^3+^ and Zn^2+^ ions, impacted both the corrosion potential and cathodic current during electrochemical polarization. Etienne et al. [69] evaluated the local corrosion phenomena, the self-healing properties of an organic/metallic coating on steel, and tracked the sealing process of nanoporous alumina anodized layers [7]. In brief, the authors observed an increase in pH during metal dissolution, followed by the formation of a protective/sealing layer, and finally the consolidation of this layer indicated by the local pH value reaching the bulk pH.

Magnesium and its alloys have also been explored in biomedical applications through these pH sensors [82]. Jamali et al. studied the degradation of AZ31 [52] and AZNd coated with Pr(NO_3_)_3_ [99] in simulated body fluid. The authors found that in the case of AZ31, the interfacial pH was highly alkaline and significantly different from the bulk pH, even in a buffered solution. On the other hand, for the Pr(NO_3_)_3_ conversion coating on AZNd, they found that Pr^+3^ acted as an effective corrosion inhibitor with self-healing characteristics, taking advantage of its dynamic deposition at highly alkaline domains. Filotás et al. [61] explored the use of a multi-barrel electrode assembly to characterize the corrosion of AZ63 magnesium alloy. The authors achieved simultaneous detection of Mg^2+^ ions and pH changes, avoiding at the same time the effect of the electric field developed around local anodes and cathodes. Tefashe et al. [70] described the local degradation of PEDOT-coated AZ31B Mg alloy. They measured the pH changes of a coated AZ31B/bare AZ31B couple. The authors noticed that the PEDOT coating gradually lost its initial protective ability due to localized coverage of corrosion products. Gnedenkov et al. [100] identified differences in the corrosion process of MA8 Mg alloy in 0.83% NaCl solution and MEM at the microscale level. The authors found that the local pH of MEM was stabilized over time by the formation of a hydroxyapatite layer, which decelerated the corrosion process. Figure 7b shows the distribution of currents and pH obtained using the scanning vibrating electrode technique (SVET) and scanning ion-selective electrode technique (SIET) on the MA8 alloy in cell culture medium, as well as the SEM-EDS information of the product layer.

There are also other metallic materials that have been examined with pH probes. Zhu et al. explored the local pH and Fe^2+^ distribution over a 316 L stainless steel surface [53,62]. They detected high concentrations of Fe^2+^ at the local anode and an increase in pH values at the local cathode. These measurements allowed tracking the formation of a stable pit over the steel surface. Ramírez-Cano et al. [101] reported the pH distribution over Cu samples treated with an organic corrosion inhibitor. The authors found that the treated Cu surface displayed more alkaline pH values than the non-treated one. This behavior was attributed to the protective effect of the inhibitor, making the treated surface act as a cathode where oxygen reduction reaction took place. Asserghine et al. [55] monitored the local pH during the self-healing of the TiO_2_ passive layer on a Ti dental implant. They observed that during the formation of the passive layer, the pH decreased and that this process was not instantaneous, as previously assumed. Da Silva et al. [54] analyzed the distribution of reactive sites and local pH change during severe localized corrosion on 2098-T351 Al alloy. They observed that at the severe localized corrosion sites, there was lower pH, higher H_2_ evolution, and lower O_2_ consumption. More recently, additive manufacturing alloys have also been studied by means of tracking local pH changes [63,102]. Meiszterics et al. [63] investigated and compared the corrosion performance of a 3D-printed AlMg_4.5_Mn_0.7_ Al alloy and its conventional counterpart as a substrate plate. They applied SECM and a W/WOx pH sensor along with conventional electrochemical measurements for such a purpose. Figure 7c presents the pH distribution over the printed material/base material boundary region and its optical image, confirming that the printed plate displayed higher corrosion resistance than the substrate material.

### 5.3. Energy and Environment

Measuring pH in fields like green energy production and environmental remediation can help to understand and tune the electrochemical processes involved, thus making them more efficient. Chemical solubility and reaction kinetics are affected by a change in pH, and electrocatalytic reactions can also induce a shift in the local pH values. The first attempts to determine pH changes at the electrode/electrolyte interface were for hydrogen evolution and oxygen reduction reactions. The hydrogen evolution reaction (HER) at noble metals has been used as a model system to test both potentiometric [40] and voltammetric [45] pH sensors. During HER, water is reduced and the generated OH^-^ ions increase the interfacial pH:

(8) $$2H_{2}O+2e^{-}\to H_{2}+{\mathrm{OH}}^{-}$$

The oxygen reduction reaction (ORR) has also been used as a standard system for pH measurements [36]. As ORR occurs, the local environment becomes more alkaline due to the production of hydroxide anions:

(9) $$O_{2}+4e^{-}+2H_{2}O\to 4{\mathrm{OH}}^{-}$$

**Figure 8:**
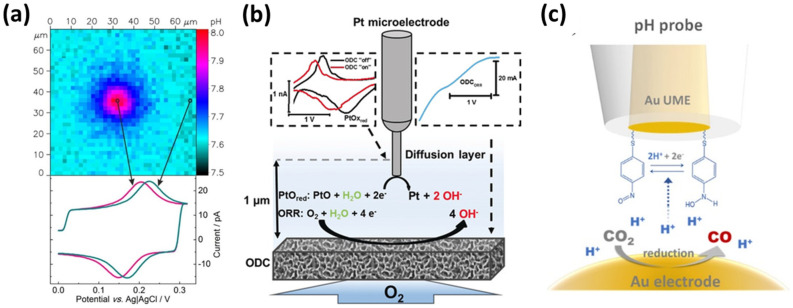
(**a**) 3D map with local pH values during ORR at a Pt UME. Adapted from ref. [36]. Copyright (2015) with permission from the American Chemical Society. (**b**) Depiction of the pH measurements at the surface of a working oxygen depolarized cathode. Reprinted from ref. [71]. Copyright (2018) with permission from John Wiley and Sons. (**c**) Scheme showing local pH measurements during CO_2_ reduction at an Au electrode. Adapted from ref. [46] under CC BY-NC-ND 4.0 license.

Figure 8a presents a 3D plot of the pH values at a Pt UME during ORR in aerated PBS, obtained using a voltammetric sensor [36]. Regarding energy applications, Ben Jadi et al. [103] analyzed the permeability resistance and proton conductivity of Nafion membranes modified with polypyrrole [104] and polyaniline [103] for direct methanol fuel cells. The authors measured local pH changes at the membrane/solution interface caused by the diffusion of H^+^ through the membrane. Compared to commercial Nafion-112, the polypyrrole-modified membrane showed a decrease in proton conductivity, whereas the polyaniline-modified membrane displayed an increase in proton conductivity.

For electrocatalysis, Botz et al. [71] investigated the local activities of OH^-^ and H_2_O in an operating oxygen depolarized cathode (ODC) as shown in Figure 8b. It was demonstrated that the H_2_O/OH^-^ activity ratio was double the value in bulk solution at 1 µm above the ODC surface during ORR. The authors suggested that this drastic change in the reaction environment resulted in the switch of the reaction inside the confined pores of the working ODC.

Monteiro et al. explored changes in pH at the diffusion layer during CO electro-oxidation [46] and CO_2_ electrochemical reduction [47,105]. In their first work, the authors noticed that the two distinct peaks from CO oxidation voltammetry were related to the diffusion limitation of CO and OH^–^ species in the pH range between 7 and 11. Figure 8c shows a schematic representation of the Au/4-NTP sensor operating during CO_2_ reduction to detect local pH changes in their second work. Their studies showed a time-dependent decay in the interfacial pH after CO_2_RR was stopped at the electrode, and how the species at the interface recovered their initial concentration, similar to the bulk solution.

## 6. Conclusions and Outlook

In summary, there has been great interest in probing the pH at the microscale, and significant progress has been achieved in the development of miniaturized, stable, and multifunctional pH probes over the last decade. Table 1 summarizes some advanced pH sensors with their respective parameters and applications, ranging from measuring pH changes in single cells to those in biofilms, from corrosion of biomedical alloys to novel 3D-printed metal parts, and from water splitting to CO_2_ reduction. The sensitivity, range, and response time of pH sensors have been notably improved. The most popular type of pH sensor is the potentiometric solid microelectrode, with different versions designed by various research groups from all over the world. Ion-selective microelectrodes based on a liquid membrane cocktail are also widely used, especially for corrosion applications. Nevertheless, potentiometric sensors display a low success rate and often need recalibration. As an alternative, voltammetric sensors have attracted more attention due to their high temporal resolution and stability of the pH response. One of the main advantages of these joint efforts is that there are a wide variety of options for fabricating a micro pH sensor, which can be tailored depending on the instrumental capabilities and final applications [106,107,108]. When integrated with scanning probe methods, these pH sensors can provide information with high spatial resolution. Single measurements, diffusion/concentration profiles, line scans, and 3D maps are examples of the typical results that can be obtained with so-called pH microscopy. In addition, some of the positioning methods, namely shear force and SICM, can concurrently provide topographical information. On the other hand, the field of micro-sized pH sensors is relatively new, and more research & development is required to: (i) improve sensor stability, especially in extreme environments; (ii) incorporate finite element modeling to account for probe effects [109]; and (iii) enhance sensor capabilities for measuring multiple features of a system in a single experiment. It is anticipated that micro-/nano-sized pH sensors will play an important role in studying the kinetics of electrochemical reactions by monitoring local pH changes, as well as in understanding the structure-activity relationship at electrode/electrolyte interfaces.

## Figures and Tables

**Table 1:** Summary of micro/nanoprobe pH sensors with their parameters/characteristics and respective applications.

Type	Material	Size (µm)	Sensitivity (mV/pH)	Range	Response Time (s)	Application	Ref.
SECMPot.	Pt/IrOx	10–25	59–75	1–12	≤1	Corrosion of stainless steel 316L	[53,60,62]
						Corrosion of PEDOT-coated AZ31 alloy	[70]
						Self-healing of organic/metallic coatings on steel	[69]
						Metabolic activity in a rat kidney slice	[110]
						Acidification kinetics at wet mineral-bacteria/air interface	[72]
	Ir/IrOx	7–20	63–70	2–13	-	Corrosion of AZ31 alloy and self-healing of AZNd alloy	[52,99]
						Corrosion at cut edges of metal-coated steel	[98]
	W/WOx	30–60	53	2–11	-	Corrosion of 3D-printed Al alloy	[63]
						Operation of DMFC with modified membrane	[103,104]
	Au/IrOx	25	74	2–11	2.5	Nitrate reduction at a Cu electrode	[43]
						Cobalt electrodeposition on brass plates	[111]
	Sb/SbOx	10–30	42–59	3–11		Corrosion of Al alloy, Mg alloy, and NiTi alloy	[54,61,112]
					<1	Galvanic coupling of Zn-Cu, Zn-Fe, and Ti-Mg	[95,96,113]
						Corrosion at cut edges of coil-coated galvanized steel	[114,115]
	C/IrOx	0.7	65	2–12	<5	Water electrolysis at an Au disk	[40]
	Pt/PANi	25	53–59	4.5–9.5	10	Bioactive glass reactivity and biofilm calcification process	[35,65]
		10				Dissolution of Fe in sulfuric acid solution.	[34]
		25				HFF, MCF-7, and HeLa cells under electrical stimulation	[28,64]
	Pt/IrOx/Nafion	25	67	1–12	-	Chromium electrodeposition at an Au substrate	[84]
SECMVolt.	Pt/PtO	25	0.03 V/dec ^a^ 0.70 V/dec ^b^	0.01–10 M ^a^ 10–20 M ^b^	CV	Oxygen depolarized cathode in operation	[71]
	Au/4-NTP	50	57	2–10	CV	HER and CO_2_ reduction on Au substrate	[45,46,47]
	C/Syr	0.05	~54	2–12	CV	ORR at a Pt substrate in PBS	[36]
	CF/rGO/Syr	37	60	2.9–7.4	CV	Extracellular spaces of cancer cells and normal cells	[37]
SICM	Au/PANi	0.34–0.6	57	2.5–14	2–12	Migration and diffusion processes at a porous PI membrane	[33]
	C/IrOx	0.1	79	2–10	-	Dissolution of calcite microcrystal	[50]
	PLL/GOx	0.1	15–20 ^c^	4–9	0.002	Pericellular environments of cancer cells	[2]
						Phycosphere of marine green algae in light/dark	[88]
ISME	H^+^ ionophore I (Tridodecylamine)	2–30	53–59	4–12.5	0.5–3	Corrosion of biomedical grade Mg alloys	[100,116,117]
						Galvanic corrosion in Al alloys	[118,119,120]
						Metabolic exchange between two bacterial species	[48]
	H^+^ ionophore II (4-nonadecyl pyridine; ETH 1907)	1.5–20	49–59	3–9	3–10	Sealing reaction at anodized Al alloy surface.	[7]
						Corrosion protection/inhibition of Al alloys	[121,122,123]
						Galvanic corrosion and corrosion fatigue of Al alloys	[124,125,126]
						Evaluation of metallic and epoxy coatings on steel	[127,128,129,130]
	3,4,5-tris (dodecyloxy)benzylbenzylethyl amine	1.5–1.8	52–55	4–13	<2	Coatings and corrosion inhibition of Al alloys	[131,132,133,134,135]

^a^ Slope from 0.01 to 10 M NaOH concentration. ^b^ Slope above 10 to 20 M NaOH concentration. ^c^ For Erev = ±0.6 V. CV (dependent on the scan rate of cyclic voltammetry).

## Data Availability

Not applicable.
